# Editorial: Atmospheric chemistry and air pollution

**DOI:** 10.1016/j.mex.2024.102588

**Published:** 2024-01-26

**Authors:** Pavlos Kassomenos

**Affiliations:** Department of Physics, University of Ioannina, University Campus, Ioannina GR-45110, Greece

Air pollution as a branch of Atmospheric Chemistry is one of the most critical human and environmental health issues we face today. WHO report attributes approximately 7 million deaths worldwide to the deleterious impacts of air pollutants. This amounts to 1 in 8 deaths across the globe, with almost 4 million deaths resulting from poor ambient air quality. Indoor air quality is also one big challenge to human health and maybe indoor air pollutants are responsible for more than 4.3 million deaths worldwide.

The link between air quality and mortality gain focus during the last twenty years, nevertheless there are also significant air pollution impacts on other sectors such as health care costs, loss of agricultural crops, materials and building damage, and damage to ecosystems.

Recently a strong correlation between the emissions of air pollutants and the greenhouse gases (GHGs) emissions was released due to the combustion of fossil fuels and biomass results not only in air pollutant emissions but also the release of carbon dioxide mainly and other GHGs into the atmosphere. The last is of utmost importance since humanity suffers from climatic crisis which increases the costs of the health benefits alone outweighed the costs of reducing the emissions of GHGs. Moreover, climatic crisis increases the number and severity of wildfires as recently happened in the Mediterranean region and especially in Greece.

The sources of air pollutants are varied. Major source categories include mobile source emissions (e.g., from vehicles, shipping, wildfires, and the air), stationary sources (power plants and industry), residential fuel combustion, biomass burning, resuspended geological material, and natural sources. Once emitted, these pollutants can undergo both transport and transformation in the atmosphere subsequent to the resultant human exposure and/or ecosystem impacts.

Significant sources are found indoors. Since people spend their time for at least 80 % of the day indoors, indoor air pollution is a significant contributor to the deterioration of the quality of life. Moreover, there are possibilities for the escape of significant substances indoors that may be dangerous for the health of the residents.

To reduce the negative effects of air pollutants, we must improve our understanding of all the factors leading to their emission, transport, transformation, and deposition/impacts. So new monitoring and assessment techniques and methodological tools need to be developed, along with the deployment of routine monitoring to implement effective strategies to control air pollution.

In the concept of this special issue more than 30 distinguished scientists worldwide were invited to contribute submitting their recent work in the disciplines of new measuring techniques and methodological tools for atmospheric chemistry and air pollution.

A few of them accepted the invitation and submitted their work.

The submitted papers were peer reviewed according to the rules of Elsevier's Methods X journal by at least two independent reviewers.

This special issue is comprised of six papers including:

Methodologies in measuring atmospheric nanoparticles charge and how their range affects human health. In this context an additional paper proposing a novel contrast index combining charged nanoparticles, climate change and public health was published.

Another paper proposing both an improvement in existing measuring technique and a methodological tool to analyze precisely persistent organic pollutants and molecular tracers in atmospheric samples was also published.

A paper proposing a direct method to quantify methanol-soluble organic carbon for Brown carbon absorption studies was selected to include in this special issue.

Finally, to cover the indoor air quality a paper was selected dealing with the dispersion of a light chemical agent (as ammonia) in a semi closed environment (office and a venue). In this paper new indexes were proposed to understand if the amount of this agent is dangerous to the health of the residents.

I hope that this Special Issue will contribute to draw to this subject the attention, it deserves considering the implications on health. Certainly not all aspects have been covered and indeed further research is needed that we hope will emerge in the near future.

We thank all the authors of the papers included in this Special Issue for their continuous support of MethodsΧ.

